# Subchondral Insufficiency Fracture of the Knee Previously Diagnosed as Spontaneous Osteonecrosis: A Case Report With Histological Analysis

**DOI:** 10.1155/cro/9109042

**Published:** 2026-07-11

**Authors:** Pierangelo Za, Giuseppe Francesco Papalia, Fabrizio Russo, Francesco Luigi Gambaro, Rocco Papalia

**Affiliations:** ^1^ Research Unit of Orthopaedic and Trauma Surgery, Fondazione Policlinico Universitario Campus Bio-Medico, Rome, Italy; ^2^ Minimally Invasive Robotic Hip and Knee Surgery Unit, IRCCS San Raffaele Hospital, Milan, Italy; ^3^ Oncological Orthopaedics Department, IFO-IRCCS Regina Elena National Cancer Institute, Rome, Italy; ^4^ Department of Orthopaedic and Trauma Surgery, Università Campus Bio-Medico di Roma, Rome, Italy, unicampus.it; ^5^ Anatomical Pathology Operative Research Unit, Fondazione Policlinico Universitario Campus Bio-Medico, Roma, Italy

**Keywords:** histological analysis, knee, osteonecrosis, SIFK, subchondral insufficiency fracture

## Abstract

**Objectives:**

Spontaneous osteonecrosis of the knee (SPONK) shares clinical and imaging similarities with subchondral insufficiency fractures of the knee (SIFKs), leading to confusion in terminology and diagnosis.

**Case Presentation:**

We reported the case of a patient with knee pain and a presumed diagnosis of SPONK based on clinical and imaging findings. X‐rays showed subchondral bone collapse and marked subchondral sclerosis. MRI showed a subchondral fracture line with extensive bone marrow edema. The lesion was classified as Stage IV according to the Koshino radiographic classification and Stage IV based on the Sayyid classification for MRI findings. The patient underwent total knee arthroplasty, during which bone samples were collected and examined. Histological analysis revealed no osteonecrosis but rather subchondral stress fractures with associated bone callus formation, proliferation of chondrocyte clusters, increased osteoblastic activity, and bone marrow edema with mild fibrosis and chronic inflammation.

**Conclusion:**

This case report highlights the discrepancies between histological findings and the current classification of osteonecrosis of the knee, suggesting that the term “osteonecrosis” may be inappropriate in some cases. Greater attention should be given to the histological substrate of these pathologies to potentially refine the nomenclature and diagnostic criteria in the future.

## 1. Introduction

Osteonecrosis (ON) of the knee was first described by Ahlbäck et al. in 1968, based solely on imaging findings without histological confirmation [1]. He described this form of necrosis as “spontaneous,” not having identified a trigger [1]. Later, subchondral insufficiency fractures of the knee (SIFKs) were identified as subchondral plate fractures with perifocal, flame‐like marrow edema [2, 3]. This has led to confusion in terminology and diagnosis due to the similarities between SIFK and spontaneous osteonecrosis of the knee (SPONK), as emphasized by various authors [2, 4–8]. SIFK results from repetitive stress and overloading in the subchondral bone, often secondary to meniscal injuries [6, 9, 10]. Clinically, SIFK presents with knee pain and effusion, which may persist even at night and is worsened by weight‐bearing activities, usually localized to the medial femoral condyle [5]. Magnetic resonance imaging (MRI) is preferred for diagnosing SIFK, although advanced lesions can also be detected on plain radiographs (X‐rays) [3, 11]. Although several authors have shown the absence of necrosis in this type of subchondral injury [12–18], the term “spontaneous osteonecrosis” is still frequently used. The histological characteristics of SIFK have been documented by MacDessi et al. [18], and the MRI features of this condition are now clearly defined [3, 11, 19]. However, even in recent literature reviews [20–22], the term SIFK is often omitted, making it more challenging to differentiate between necrotic lesions and subchondral fractures. In our opinion, beyond semantic differences, it is crucial to understand the histological basis underlying these types of subchondral lesions in order to better correlate imaging characteristics with histopathological findings. This approach would help establish accurate terminology, improve the understanding of the pathology, and ultimately lead to a proper diagnosis, which can guide effective and standardized treatment strategies. A potential definitive shift from a necrotic to a subchondral fracture–based pathogenesis would lead to new interpretations of MRI findings and could open the door to novel treatment strategies. Therefore, our aim was to examine the histological features of bone removed from a patient who underwent total knee arthroplasty (TKA) following a diagnosis of SPONK.

## 2. Case Report

A 75‐year‐old woman with a body mass index (BMI) of 35.5 presented to us on January 29, 2024, complaining of a 1‐month history of gradually worsening left knee pain, which occurred in the absence of any trauma and was exacerbated by weight‐bearing. Informed consent was obtained from the participant included in the study (Table 1). Her medical history revealed no significant comorbidities, home treatments, or known allergies. She had no history of recent fever, corticosteroid (CS) use, smoking or drug abuse, alcohol intake, or any recent knee injuries. The patient was not taking any regular medications since she was not suffering from any known pathology. Clinical examination of the left knee showed a range of motion (ROM) from 10° to 90°, with pain at maximum flexion and during attempted full extension. There was no increased warmth upon palpation, but tenderness was noted over the medial joint line and the medial femoral condyle. A positive patellar tap test suggested the presence of joint effusion. An arthrocentesis was performed, yielding serous fluid. Based on the clinical history and examination findings, a diagnosis of knee osteoarthritis was suspected. The patient was advised to pursue conservative treatment for a duration of 1 month consisting of etoricoxib 60 mg once daily for 2 weeks, weight‐bearing as tolerated without the use of crutches, and a physiotherapy program focused on isometric strengthening of the lower limb musculature, with particular emphasis on the quadriceps and gluteus medius muscles, combined with stretching exercises.

**Table 1:** Patient’s history timeline.

Date	Event
January 29, 2024	Initial presentation with a 1‐month history of progressively worsening atraumatic left knee pain. Clinical examination was performed, and conservative treatment was initiated for suspected knee osteoarthritis.
May 27, 2024	Plain radiographs demonstrated medial compartment osteoarthritis associated with subchondral collapse of the medial femoral condyle.
May 28, 2024	MRI revealed advanced medial compartment degeneration, severe degenerative medial meniscal pathology with extrusion, osteochondral/subchondral lesion, and extensive bone marrow edema–like signal.
June 2024	Follow‐up evaluation showed persistent symptoms despite conservative management. A diagnosis of advanced SPONK/SIFK was established, and the lesion was considered no longer reversible. Surgical treatment was, therefore, indicated.
August 2024	Total knee arthroplasty was performed, and histological sampling of the lesion was obtained for pathological evaluation.

### 2.1. Imaging Acquisition

Further evaluation was recommended, including full‐length standing AP and lateral view X‐ray, Rosenberg views, and Merchant view of the patella, as well as a 1.5 T MRI scan. The patient returned for evaluation in June 2024, reporting persistent symptoms despite the early management. X‐rays and MRI studies of the left knee were presented for review. X‐rays (May 27, 2024) showed osteoarthritic changes characterized by sclerosis of the articular surfaces and a reduction in joint space, most pronounced in the medial femorotibial compartment (Figure 1). A semilunar region of subchondral bone collapse was identified in the weight‐bearing zone of the medial femoral condyle, surrounded by a distinct sclerotic margin. According to the Koshino classification, the lesion was consistent with Stage IV disease, as demonstrated by radiographic findings of subchondral bone collapse, marked subchondral sclerosis, significant joint space narrowing, and secondary osteoarthritic changes involving the femorotibial compartment.

**Figure 1:**
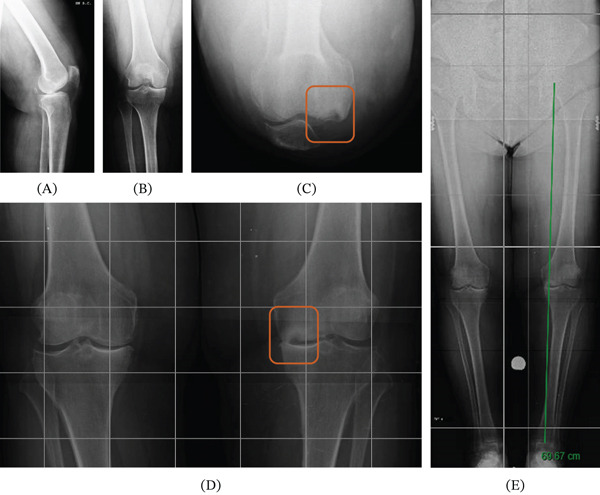
(A) Lateral view, (B) Rosenberg views, and (C) Merchant view of the patella of the left knee. (D) Frontal view of the right and left knee and (E) full‐length X‐rays of the lower limbs. (C, D) The semilunar region of subchondral bone collapse is visible in the weight‐bearing zone of the medial femoral condyle, surrounded by a distinct sclerotic margin (highlighted in orange).

MRI (May 28, 2024) revealed advanced degenerative changes predominantly affecting the medial femorotibial compartment. MRI was performed using T1‐weighted, T2‐weighted, and fat‐suppressed proton density (PD‐FS) sequences acquired in axial, coronal, and sagittal planes. No contrast was administered. Imaging was reviewed by a musculoskeletal radiologist. MRI interpretation was based on standard clinical radiological criteria for subchondral insufficiency fractures and advanced osteoarthritic degeneration. MRI findings fulfilled criteria for Sayyid Stage IV [16], characterized by extensive bone marrow edema–like signal involving the weight‐bearing portion of the femoral condyle measuring 21 mm in the anteroposterior dimension, 24 mm mediolaterally, and 6 mm in depth, involving the majority of the weight‐bearing surface of the distal medial femoral condyle, associated with a subchondral fracture line and advanced structural joint degeneration (Figure 2). These combined features were consistent with a late‐stage subchondral insufficiency fracture in an advanced degenerative setting. There was evidence of a complex medial meniscal tear with a meniscal flap and approximately 7 mm of meniscal extrusion. Severe degenerative meniscal pathology involving the posterior horn and root of the medial meniscus was present, although no frank posterior medial meniscal root tear was identified. Additionally, a monocondylar osteochondral lesion in the epiphyseal area with bone marrow edema–like signal intensity was identified.

**Figure 2:**
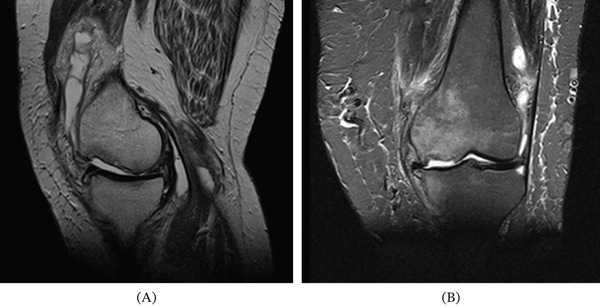
(A, B) MRI of the left knee revealed advanced degenerative changes predominantly affecting the medial femorotibial compartment. Medial meniscal tear with meniscal extrusion. Additionally, a monocondylar osteochondral lesion in the epiphyseal area with bone marrow edema–like signal intensity is shown.

### 2.2. Treatment

Based on the clinical and radiological findings, a diagnosis of SPONK was established. The lesion was classified as Stage IV according to the Koshino radiographic classification [23] and as Stage IV based on Sayyid et al.′s [19] classification for MRI findings. Given the poor prognosis associated with advanced lesions [9, 24–27], characterized by subchondral collapse and advanced osteoarthritis, the decision was made to proceed with TKA. Joint‐preserving procedures were not considered, as current literature suggests limited effectiveness and high failure rates in advanced‐stage disease. TKA was preferred over unicompartmental knee arthroplasty (UKA) because of the patient′s markedly elevated BMI. A TKA was performed in August 2024 (Figure 3).

**Figure 3:**
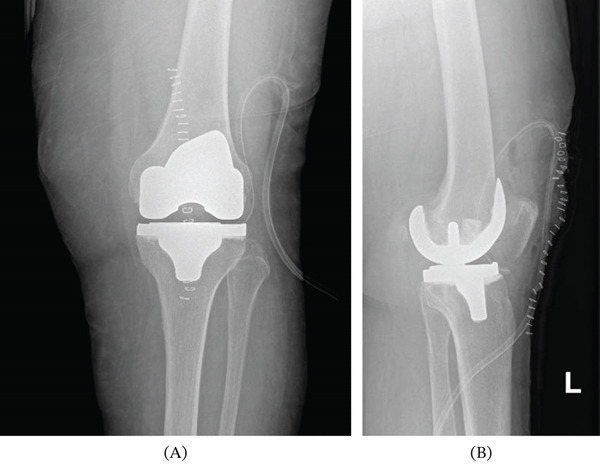
(A) Frontal and (B) lateral views of total knee arthroplasty.

During the procedure, a large bone sample measuring approximately 9 mm in thickness was harvested from the medial femoral condyle at the site of the osteochondral lesion, following the bone resection. The sample was subsequently sent for histological examination. The surgical procedure was carried out without complications. The postoperative clinical course was unremarkable. The patient underwent physiotherapy without complications and was seen 2 weeks after surgery for stitch removal and 1 month after surgery for clinical follow‐up. At 1 month postoperatively, the patient reported resolution of pain and was clinically improving; however, no validated functional outcome score was available.

### 2.3. Histology

The bone specimen obtained from the affected femoral condyle during knee arthroplasty was fixed in 10% neutral buffered formalin for approximately 24–48 h and processed according to standard musculoskeletal histopathology protocols. The decalcification procedure was performed. The specimen was paraffin‐embedded, and histological evaluation was performed using hematoxylin and eosin (H&E) staining. One tissue block corresponding to the lesion area was examined. The analyzed specimen corresponded to the prosthetic bone resection and measured approximately 9 mm in thickness. The entire specimen, from the articular surface to the resection margin, was assessed histologically. No spatial mapping between histological and radiological findings was performed. Histological evaluation was jointly reviewed by an orthopedic surgeon and a musculoskeletal pathologist, with the latter blinded to the clinical and radiological diagnosis. Microscopic evaluation was performed using standard light microscopy at ×10 and ×20 magnification. ON was excluded based on the absence of characteristic histopathological findings, including empty osteocyte lacunae, diffuse osteocyte loss, marrow necrosis, necrotic trabecular bone, and reparative granulation tissue surrounding necrotic areas.

Histological examination (Figure 4A–E) of the whole specimen showed loss of articular cartilage with pannus formation and diffuse alterations involving subchondral bone and consisting mainly of cortical bone microfractures with associated bone callus formation, proliferation of chondrocyte clusters, increased osteoblastic activity, and bone marrow edema with mild fibrosis and chronic inflammation. Such findings were focal and neatly demarcated from surrounding undamaged cartilage. No ON, understood as a true bone infarction [28], was detected. Histological findings were consistent with subchondral bone collapse and advanced degenerative joint disease. In consideration of clinical history and radiology findings, they were deemed compatible with late‐stage SIFK.

**Figure 4:**
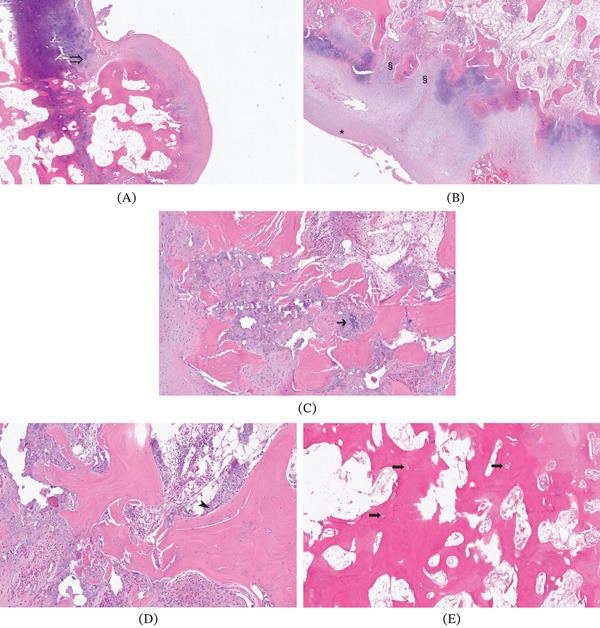
(A) Lesion was neatly demarcated. Black arrow shows a sharp limit between mostly spared articular cartilage (upper side) and damaged, hyperproliferating articular cartilage (bottom side) with underlying sclerosing bone trabeculae. (B) Pannus formation (∗) accompanied the aforementioned hypercellularity, matrix depletion of proteoglycan, and focal loss of mineralization even in the basal layer (§). (C) Subchondral bone fragmentation (white arrows) and chondrocytic proliferation foci (black arrow) were evident. (D) Bone trabeculae fragmentation was partially accompanied by increased osteoblastic activity (arrow) and bone sclerosis. (E) No bone necrosis was observed, as shown by the regular presence of osteocytic nuclei in their respective lacunae (black arrow).

## 3. Discussion

This case report describes the clinical course of a patient initially diagnosed with presumed SPONK based on clinical and imaging features. Our patient presented with the typical clinical features of SPONK, and this led us to make a diagnosis of necrosis on the basis of old classifications [21]. The symptomatology of SPONK is characteristic but extremely similar to that reported for SIFK [1, 5, 21]. The gold standard for diagnosis is MRI [5, 19], which can detect early lesions. In contrast, X‐rays often fail to detect early lesions and only become positive in advanced stages of the disease. Early diagnosis by MRI is crucial, as the prognosis is directly proportional to the stage of the lesion [9, 24, 25, 27]. In the early stages, conservative treatment is effective and mainly includes physical therapy, weight protection, bisphosphonates, electromagnetic fields, anticoagulants, and hyperbaric oxygen therapy [29]. However, in advanced stages, conservative therapy loses its effectiveness due to the collapse of the articular surface. In this context, several treatments aimed at preserving the joint have been described, including core decompression, tibial osteotomy, and repair of the osteochondral defect [21]. However, these solutions are often ineffective in the long term, and prosthetic replacement is often necessary [30]. Our histological examination excluded necrosis and instead revealed typical findings of subchondral insufficiency fractures, confirming the initial misnomer and, thus, the misdiagnosis. At the end of the diagnostic work‐up, a diagnosis of degenerative primary SIFK was established based on the integration of clinical history, physical examination, and imaging findings. In this context, both the associated meniscal pathology and increased BMI were considered plausible triggering factors contributing to disease onset and progression. This diagnostic hypothesis was subsequently supported and strengthened by histological analysis, which confirmed the absence of ON. This confirms the current challenges in differentiating between ON and SIFKs, as well as the lack of consistency in associating specific clinical and imaging characteristics with a definitive diagnosis. This issue is further compounded by the absence of an updated nomenclature, leading to confusion between SIFK and true avascular conditions.

The current classification [21] recognizes three types of ON of the knee: spontaneous, postarthroscopic, and secondary ON. Spontaneous ON was first described by Ahlbäck et al. [1] in 1968 as a condition characterized by the sudden onset of severe, persistent pain, especially at night, localized to the medial femoral condyle within the weight‐bearing area, without an apparent cause. On radiographs, SPONK typically presented as subchondral radiolucency, flattening of the articular surface, and an oval‐shaped bone defect [23]. Subsequently, MRI revealed the lesion as a monocondylar abnormality in the epiphyseal region with bone marrow edema–like signal intensity, often extending throughout the condyle [3, 11]. Despite being termed “spontaneous osteonecrosis,” there was no histological evidence of necrosis reported by Ahlbäck et al. [1], as the criteria applied for the diagnosis of ON were derived from imaging characteristics of ONs at other joints, such as the hip. Later studies by Yamamoto and Bullough [2] demonstrated the absence of necrosis in histological samples from patients with the classic clinical and imaging findings of SPONK. Numerous subsequent studies confirmed these observations [12–17], revealing subchondral fractures rather than necrosis in patients diagnosed with knee ON. Koshino [31] identified small, localized necrotic areas associated with subchondral fractures in advanced cases, but these should not be misinterpreted as true ON [2, 5, 32, 33]. Several theories have been proposed regarding the mechanisms underlying the onset of necrosis [21, 24, 34]; however, the most recent theory suggests that small foci of necrosis may arise from the failure of healing attempts in subchondral fractures, which subsequently triggers cellular death [7, 12, 32]. This perspective emphasizes the role of compromised bone healing processes in the development of necrotic lesions, highlighting a potential link between mechanical stress and necrotic changes in the affected tissues. In this case report, MRI findings demonstrated meniscal extrusion, and severe meniscal degeneration was confirmed intraoperatively. This finding supports the well‐established cause–effect relationship between meniscal pathology, particularly posterior meniscal root tears associated with meniscal extrusion, and the progression of subchondral lesions [35]. It is conceivable that, also in this case, an unrecognized and untreated meniscal lesion may have contributed to the progression of the disease, ultimately leading to an irreversible condition. In our reported case, despite the advanced stage of the lesion, no signs of necrosis were identified. Similar histological results were also observed for postarthroscopic osteonecrosis of the knee (PONK). Madry et al. [34] conducted a subsequent analysis of samples collected from seven patients (eight knees) who had knee arthroplasty following a diagnosis of ON. His findings revealed that necrosis was present in only two of the knees, while all the remaining knees exhibited subchondral fractures accompanied by bony callus formation. Therefore, the term PONK was questioned too [6, 8]. This led to the recognition of SIFK as a distinct entity, with some authors advocating for a change in terminology, replacing the term “spontaneous osteonecrosis” with SIFK. This raises the possibility that Ahlbäck et al.′s original description in 1968 [1] was, in fact, SIFK, mistakenly identified as ON. However, it is important to distinguish SIFK from secondary osteonecrotic lesions. Secondary ON of the knee, initially described by Ahlbäck [36], is associated with risk factors such as smoking [37], drug abuse [38], alcohol consumption [39], CS use [39, 40], hematologic disorders [41], viral infections [42], and autoimmune diseases [43–45]. Unlike SIFK, secondary ON is a true avascular necrosis with histological evidence of cell death. It has specific clinical and imaging features that differentiate it from SIFK [21]. However, some limitations must be noted. First of all, the histological conclusion of “no osteonecrosis” is based on a limited surgical sample from the medial femoral condyle. Sampling bias cannot be excluded, as focal necrotic areas elsewhere within the lesion may have been missed. Therefore, the absence of necrosis should be interpreted as the absence of established ON in the examined tissue rather than the definitive exclusion of necrosis throughout the entire lesion. Secondly, while the presence of osteocyte nuclei in lacunae argues against established ON, early or evolving necrosis may not yet show complete osteocyte loss. In addition, it describes a single patient, thereby limiting the generalizability of the findings. The lesion was evaluated at an advanced stage of disease, in the presence of concomitant osteoarthritis, which may have influenced both imaging and histological interpretation.

Furthermore, no systematic spatial correlation between MRI findings and histological sections was performed. Nevertheless, some strengths can be identified. A major strength of this case report compared with the existing literature is the demonstration of a clinicoradiological–histological correlation in an advanced lesion (Koshino Stage IV/Sayyid Stage IV), with histological findings consistent with late‐stage subchondral insufficiency fracture and no evidence of established ON in the sampled tissue. The histological analysis of tissue obtained during TKA allows direct assessment of the subchondral lesion in an advanced‐stage setting. Finally, the case contributes to the ongoing debate regarding the appropriateness of the SPONK/SIFK terminology, supporting the growing evidence that some lesions traditionally classified as SPONK may instead represent advanced subchondral insufficiency fractures without established ON.

In conclusion, this case demonstrates a discrepancy between a clinicoradiological diagnosis of advanced SPONK and histological findings in the sampled tissue that were consistent with subchondral insufficiency fracture without established ON. These findings support the need for careful clinicoradiological and histological correlation when using the term SPONK, particularly in advanced medial compartment disease associated with meniscal pathology and subchondral collapse. Further studies using systematic histological sampling and standardized imaging correlation are required in support of these findings.

## Funding

No funding was received for this manuscript.

## Ethics Statement

All procedures performed in the study were in accordance with the ethical standards of the national research committee and with the 1964 Helsinki Declaration.

## Consent

Informed consent was obtained from all individual participants included in the study.

## Conflicts of Interest

The authors declare no conflicts of interest.

## Supporting information


**Supporting Information** Additional supporting information can be found online in the Supporting Information section. CARE Checklist—CARE Case Report Guidelines.

## Data Availability

The data that support the findings of this study are available on request from the corresponding author. The data are not publicly available due to privacy or ethical restrictions.
