# Supplementary material for: Subchondral Insufficiency Fracture of the Knee Previously Diagnosed as Spontaneous Osteonecrosis: A Case Report With Histological Analysis
Source: Case Rep Orthop. 2026 Jul 11;2026:9109042. doi: 10.1155/cro/9109042 (PMC13355289; doi:10.1155/cro/9109042)
Supplement: Supplementary file 1 — Supporting Information Additional supporting information can be found online in the Supporting Information section. CARE Checklist—CARE Case Report Guidelines. [file CRO-2026-9109042-s001.pdf]

CARE case report guidelines

CARE Checklist of information to include when writing a case report

(cc) BY-NC-ND

Topic	Item	Checklist item description	Reported on Line
Title	1	The diagnosis or intervention of primary focus followed by the words "case report"	1-2
Key Words	2	2 to 5 key words that identify diagnoses or interventions in this case report, including "case report"	48
Abstract (no references)	3a	Introduction: What is unique about this case and what does it add to the scientific literature?	30-32
	3b	Main symptoms and/or important clinical findings	33-34
	3c	The main diagnoses, therapeutic interventions, and outcomes	34-41
	3d	Conclusion—What is the main "take-away" lesson(s) from this case?	42-45
Introduction	4	One or two paragraphs summarizing why this case is unique (**may include references**)	57-81
Patient Information	5a	De-identified patient specific information.	91-93
	5b	Primary concerns and symptoms of the patient.	91-93
	5c	Medical, family, and psycho-social history including relevant genetic information	94-97
	5d	Relevant past interventions with outcomes	94-97
Clinical Findings	6	Describe significant physical examination (PE) and important clinical findings.	97-101
Timeline	7	Historical and current information from this episode of care organized as a timeline	192
Diagnostic Assessment	8a	Diagnostic testing (such as PE, laboratory testing, imaging, surveys).	109-129
	8b	Diagnostic challenges (such as access to testing, financial, or cultural)	N.A.
	8c	Diagnosis (including other diagnoses considered)	137-153
	8d	Prognosis (such as staging in oncology) where applicable	N.A.
Therapeutic Intervention	9a	Types of therapeutic intervention (such as pharmacologic, surgical, preventive, self-care)	173-176
	9b	Administration of therapeutic intervention (such as dosage, strength, duration)	181-184
	9c	Changes in therapeutic intervention (with rationale)	181-184
Follow-up and Outcomes	10a	Clinician and patient-assessed outcomes (if available)	186-188
	10b	Important follow-up diagnostic and other test results	184-188
	10c	Intervention adherence and tolerability (How was this assessed?)	173-176
	10d	Adverse and unanticipated events	184-188
Discussion	11a	A scientific discussion of the strengths AND limitations associated with this case report	297-328
	11b	Discussion of the relevant medical literature **with references**.	248-261
	11c	The scientific rationale for any conclusions (including assessment of possible causes)	330-340
	11d	The primary "take-away" lessons of this case report (without references) in a one paragraph conclusion	342-348
Patient Perspective	12	The patient should share their perspective in one to two paragraphs on the treatment(s) they received	N.A.
Informed Consent	13	Did the patient give informed consent? Please provide if requested	Yes ☒ No ☐
